# Cost-consequence analysis of a combined COVID-19/influenza rapid diagnostic test in the Brazilian private healthcare setting

**DOI:** 10.1016/j.bjid.2024.103840

**Published:** 2024-07-09

**Authors:** Julia Lowin, Michelle Sotak, Laura Haas, David Wastlund

**Affiliations:** a Vista Health Pte Ltd, Singapore; b Abbott, Rapid Diagnostics Division, Abbott Park, IL USA; c Abbott, Rapid Diagnostics Division, São Paulo, SP, Brazil

**Keywords:** Influenza, COVID-19, Diagnostic testing, Costs, Resource utilization

## Abstract

Combination COVID-19/influenza rapid tests provide a way to quickly and accurately differentiate between the two infections. The goal of this economic evaluation was to assess the cost and health benefits of a combination COVID-19/influenza Rapid Diagnostic Test (RDT) vs. current standard-of-care in the Brazilian private healthcare setting. A dual decision tree model was developed to estimate the impact of rapid differentiation of COVID-19 and influenza in a hypothetical cohort of 1,000 adults with influenza-like illness in an ambulatory healthcare setting. The model compared the use of a combination COVID-19/influenza RDT to Brazil standard diagnostic practice of a COVID-19 RDT and presumptive influenza diagnosis. Different levels of influenza prevalence were modeled with co-infection estimated as a function of the COVID-19 prevalence. Outcomes included accuracy of diagnosis, antiviral prescriptions and healthcare resource use (hospital bed days and ICU occupancy). Depending on influenza prevalence, considering 1,000 patients with influenza-like illness, a combination RDT compared to standard practice was estimated to result in between 88 and 149 fewer missed diagnoses of influenza (including co-infection), 161 to 185 fewer cases of over-diagnosis of influenza; a 24 to 34% reduction in hospital bed days and a 16 to 26% reduction in ICU days. In the base case scenario (20% influenza, 5% COVID-19), the combination RDT was estimated to result in cohort cost savings of $99. Based upon a *de novo* economic model, this analysis indicates that use of a combination RDT could positively impact influenza antiviral prescriptions and lower healthcare resource use.

## Introduction

The COVID-19 pandemic illustrated how rapid tests can provide significant clinical and public health benefits for respiratory illnesses due to their low cost, fast time to results, and ease of use. Rapid tests have been available for influenza for many years, however, the relatively low performance of earlier generations of tests, particularly low sensitivity, has limited their use.^1^ Presumptive diagnosis or clinical judgment is often relied on to make an influenza diagnosis; several studies have indicated that clinical judgment alone has low diagnostic sensitivity.^2^^,^^3^

COVID-19 and influenza have overlapping symptoms,^4^ making diagnosis of individuals presenting with Influenza-Like Illness (ILI) challenging. A systematic review and meta-analysis reported that 19 % of SARS-CoV-2 patients were coinfected with another virus or bacterium, and that influenza A, influenza B, and Respiratory Syncytial Virus (RSV) were the most common viral coinfections.^5^ Distinguishing between the two viruses has become even more important with the introduction of effective COVID-19 antiviral treatments, as both COVID-19 and influenza antivirals are more effective when initiated early in the infection.^6^, ^7^, ^8^ In addition, coinfection of COVID-19 and influenza results in poorer outcomes.^9^, ^10^, ^11^

Although influenza rates decreased during the height of the COVID-19 pandemic, it is highly likely that the viruses will continue co-circulating during global respiratory virus seasons and that they will appear in unexpected ways. In Brazil, for example, an unexpected spike in H3N2 influenza A cases was seen from late 2021 to early 2022 (i.e., summer in the Southern hemisphere), out of alignment with the typical seasonal appearance in the area.^12^^,^^13^

Combination COVID-19/influenza Rapid Diagnostic Tests (RDTs) have recently become available in several countries, providing a way to quickly and accurately diagnose between the two infections. Previous economic analyses have demonstrated the clinical and health benefits of timely diagnosis of influenza and COVID-19,^14^, ^15^, ^16^ but no published models were identified that examined the benefits of simultaneously testing for both infections. The goal of this economic evaluation was to assess the cost and health benefits of a combination COVID-19/influenza RDT vs. usual practice in the Brazilian private healthcare setting.

## Materials and methods

### Decision problem

The population consisted of adults with ILI presenting at ambulatory care centres in Brazil, modeled under different scenarios of expected influenza (15 %, 20 % and 25 %) and COVID-19 (5 % and 10 %), to reflect potential levels of underlying disease in an ILI cohort. The influenza upper limit of 25 % reflects the peak of the January 2022 H3N2 outbreak in Brazil.^13^ The base case analysis assumed 20 % underlying influenza prevalence combined with 5 % COVID-19.

The intervention in this analysis is a combination antigen RDT for COVID-19, influenza A and influenza B (Panbio™ Flu/COVID-19 Rapid Panel, Abbott Rapid Diagnostics Jena GmbH). Studies have shown that presumptive clinician diagnosis is standard practice for influenza;[2,3] therefore, the comparator used in this setting was usual care defined as a COVID-19 RDT and clinical judgment for influenza (i.e., no diagnostic testing for influenza).

Outcomes included accuracy of diagnosis and expected Health Care Resource Utilization (HCRU), consisting of hospital bed days and Intensive Care Unit (ICU) occupancy. An additional analysis explored inappropriate use of antibiotics. Outcomes are reported at the cohort level and are presented as disaggregated outcomes (i.e., as a cost-consequence analysis). A Brazilian private health insurer perspective was used for the analysis.

### Economic model

A dual decision tree model was developed in Microsoft Excel to capture multiple outcomes and track parallel testing for COVID-19 and influenza in this population.

A structured literature search was carried out in PubMed to identify existing economic evaluations of COVID-19 and influenza diagnostic tests to inform the model structure. Studies were included if they reported a simple replicable model; more complex simulation models were not reviewed. Four studies/model structures were shortlisted for review.^17^, ^18^, ^19^, ^20^ These provided clear structures for test-treat modeling in COVID-19 and influenza; however, no studies were retrieved which tracked dual diagnosis in an ILI population, a key component of evaluating the use of a dual RDT. Therefore, a *de novo* model was developed incorporating the ability to include diagnoses for both COVID-19 and influenza. The key premise of the analysis followed the published literature, in that timely antiviral treatment is assumed to positively impact subsequent resource utilization. Following You et al. (one of the only models identified that evaluated the ambulatory setting),^19^ resource utilization included hospitalizations, ICU admissions, and associated length of stay. The costs of antiviral medications were not included in this analysis.

**Fig. 1:**
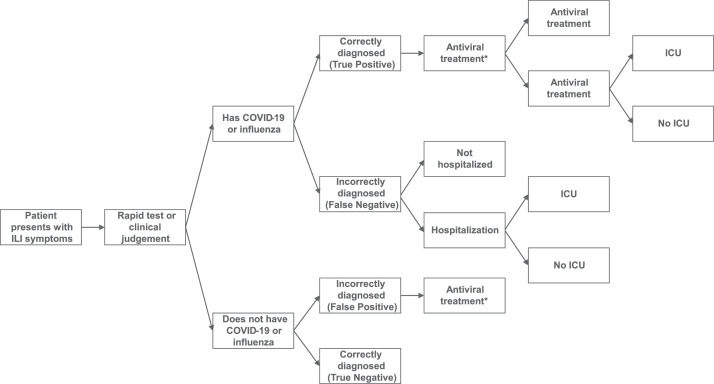
Model Structure.

The model structure is provided in Fig. 1. Individuals are classified according to their true disease status and results of the combined RDT or standard practice diagnosis. ILI patients can have influenza, COVID-19, COVID-19/influenza co-infection, or neither disease (ILI only). Outcomes are dependent on the accuracy of the test/clinical judgment and underlying disease status. Patients with a positive diagnosis (true or false) receive antiviral treatment (assumed not to be reimbursed under private insurance) while patients with a negative diagnosis do not receive treatment. The receipt of timely treatment is assumed to reduce the likelihood of resource utilization and associated costs. This assumption is in line with the approach taken in previous models.^17^, ^18^, ^19^, ^20^

*Antibiotic use was not included in the base case scenario but was assessed in an additional analysis. Abbreviations: COVID-19, Coronavirus Disease 2019, ICU, Intensive Care Unit, ILI, Influenza-Like Illness.

Although the intervention combination RDT provides separate test results for influenza A and influenza B, as influenza A accounted for the majority of cases in Brazil in 2022,^21^ a simplifying assumption was made to only consider influenza A in the analysis.

### Model inputs

Model inputs were defined based on a pragmatic review of the literature. Local data for Brazil were used where available, followed by region-specific data where possible. Table 1 outlines the parameter base case values and ranges used in the sensitivity analyses. The base case analysis assumed an influenza prevalence of 20 % and a COVID-19 prevalence of 5 %.

**Table 1:** Model parameters for base case analysis.

Parameter	Value	Range	References
Testing			
Combination RDT, sensitivity – influenza A	0.92	0.808, 0.978	Abbott ^22^
Combination RDT, specificity – influenza A	1.00	0.982, 1.00	Abbott ^22^
Combination RDT, sensitivity – COVID-19	0.88	0.80, 0.936	Abbott ^22^
Combination RDT, specificity – COVID-19	1.00	0.80, 1.00	Abbott ^22^
COVID-19 antigen test – sensitivity^a^	0.88	0.80, 0.936	Abbott ^22^
COVID-19 antigen test – specificity^a^	1.00	0.80, 1.00	Abbott ^22^
Clinical judgment – influenza -sensitivity	0.36	0.288, 0.432	Dugas et al., 2015 ^2^
Clinical judgment – influenza - specificity	0.78	0.624, 0.936	Dugas et al., 2015 ^2^
Disease management ‒ influenza			
Probability of hospitalization if untreated	0.064	0.051, 0.078	Crépey et al., 2020 ^24^
Probability of ICU stay if hospitalized	0.108	0.086, 0.130	Piroth et al., 2021 ^32^
Duration of hospitalization (days)	4.493	3.595, 5.392	Crépey et al., 2020 ^24^
Duration of ICU stay (days	8.000	6.400, 9.600	Piroth et al., 2021 ^32^
Relative risk of hospitalization if treated	0.35	0.17, 0.75	You et al., 2017 ^19^
Relative risk of ICU stay if treated	0.35	0.17, 0.75	You et al., 2017 ^19^
Mortality – hospitalized	0.058	0.046, 0.070	Piroth et al., 2021 ^32^
Mortality ‒ ICU	0.180	0.144, 0.216	Piroth et al., 2021 ^32^
Disease management – COVID-19			
Probability of hospitalization if untreated	0.190	0.152, 0.228	Hui, et al. 2021 ^33^
Probability of ICU stay if hospitalized^b^	0.230	0.184, 0.276	Crépey et al., 2020 ^24^
Duration of hospitalization (days)^c^	5.58	4.47, 6.70	Calculated
Duration of ICU stay (days)^c^	9.94	7.95, 11.93	Calculated
Relative risk of hospitalization if treated	0.49	0.46, 0.53	Hammond et al., 2022 ^6^
Relative risk of ICU stay if treated	0.35	0.17, 0.75	You et al., 2017 ^19^
Mortality – hospitalized^c^	0.092	0.073, 0.110	Calculated
Mortality – ICU^c^	0.284	0.227, 0.341	Calculated
Costs (USD^d^)			
Abbott diagnostic	$7.60	6.08, 9.12	Data on file
Generic c19 diagnostic	$3.80	3.04, 4.56	Data on file
Hospitalization – influenza (per day)	$40.46	32.38, 48.57	SUS database ^34^
ICU – influenza (per day)^e^	$141.50	113.20, 169.80	Calculated
Public: Private inflator for SUS-based costs	2.8	2.24, 3.36	Crepey et al. 2020 ^24^
Hospitalization – COVID-19 (per day)	$297.53	238.02, 357.04	Rocha et al., 2023 ^25^
ICU – COVID-19 (per day)^e^	$969.72	775.78, 1163.66	Rocha et al., 2023 ^25^

Notes.

a COVID-19 test performance is assumed equal for the panel test and individual COVID-19 test.

b High-risk influenza group used as a proxy for hospitalized COVID-19.

c LOS and rates of mortality are estimated as a function of the influenza LOS and mortality rates reported in Table 2 using the ratio of influenza: COVID-19 LOS/mortality reported in Santos et al. 2020^35^ (1.24 and 1.58 respectively).

d Converted using 1 USD: 5 BR (exchange rate, November 2023).

e ICU costs are estimated based on the ratio of total stay cost for IP:ICU reported in Miethke-Morais et al. 2021^36^ (1.79) (the ratio relates to COVID-19 costs but is assumed reflective of IP/ICU split across both diseases.

Inputs for test accuracy were taken from the combination diagnostic test package insert and published sources for presumptive diagnosis.^2^^,^^22^ The base case analysis assumed that diagnostic accuracy for COVID-19 RDTs were identical across the individual COVID-19 test and COVID-19/influenza combination test, effectively modeling the impact of testing vs. clinical judgment for influenza. No confirmatory testing was assumed for either COVID-19 or influenza.

Inputs for disease management were estimated from the literature and are reported separately for influenza and COVID-19. Co-infection inputs were estimated as a function of COVID-19 based on data reported in Alosaimi et al.^23^ where the likelihood of mortality for coinfection was estimated at 1.78 times that of COVID-19.

Unit costs for influenza hospitalizations and ICU stays were estimated in local currency using costs from the Brazilian public healthcare system (SUS) as a benchmark with an inflator used to approximate the private insurance market. This inflator was set to 2.8 based on the default used in a recent model with a private insurance perspective in Brazil.^24^ Unit costs for COVID-19 hospitalizations and ICU stays were obtained from a recent study reporting Brazilian private payer costs.^25^ Costs for influenza antiviral treatment were not included as most oral medications are not reimbursed by Brazilian private insurance plans. Costs were estimated over a short-term time horizon reflecting the acute nature of COVID-19 or influenza infection and are reported in 2022 US dollars (using an exchange rate of 0.2 USD to 1 BRL).^26^ No discounting was applied.

Scenario analysis was performed to assess the impact of underlying influenza and COVID-19 prevalence on the model outcomes and to simulate situations where influenza peaks coincide with an increase in COVID-19. This was particularly important given the seasonal variation to influenza prevalence, and that the relative prevalence of COVID-19 compared to influenza for future seasons is uncertain. Influenza prevalence was varied between 15 % and 25 % and COVID-19 prevalence varied between 5 % and 10 %.

The impact on antibiotic prescribing was also explored, as prior studies have indicated that rapid testing may reduce inappropriate antibiotic use (i.e., antibiotics in influenza-positive individuals).^27^, ^28^, ^29^, ^30^, ^31^ A simple risk reduction was calculated from a systematic review of the impact of influenza rapid testing on antibiotic use[29] and combined with an estimate of baseline levels of antibiotic prescribing to explore the impact of rapid testing on antibiotic use for influenza. Antibiotic prescription costs were included as a proxy for potential patient-out-of-pocket expenses.

### Sensitivity analyses

One-Way Sensitivity Analyses (OWSA) were conducted on key model inputs to test whether the model outputs were sensitive to variations in model inputs. Parameter values were varied by ±20 %, unless logically bounded or sources clearly specified the 95 % Confidence Intervals. Results are reported as a tornado diagram in the supplementary appendix.

The diagnostic testing market in Brazil has been very dynamic, with multiple manufacturers and rapidly changing pricing. The impact of the relative pricing of combination RDT (compared to COVID-19 RDT) was explored to look at the impact of potential fluctuations.

## Results

### Base case results

The results of the base case analysis are reported in Table 2. Use of the combination RDT resulted in reductions in over-diagnosis (false positives; 0 in the combination RDT arm vs. 174 in the standard practice arm) and missed diagnosis (false negatives) (22 for combination RDT vs. 139 for standard practice). Hospital and ICU days were lower in the combination testing arm than the standard practice arm, due to fewer false negative diagnoses of influenza. The combination RDT arm resulted in 53.2 hospital days per 1000 people tested compared to 78.8 days in the standard practice arm, or a relative reduction of 32 %. ICU days decreased from 14.99 in the standard practice arm to 10.3 in the combination RDT arm, or a 24 % relative reduction.

**Table 2:** Base case results.

Parameter	Combination Test	Usual Care	Difference
Outcomes			
True Diagnosis	243.23	117.77	125.46
Over Diagnosis	0.00	174.05	−174.05
Missed Diagnosis	22.35	138.60	−116.25
Hospital days	53.21	78.81	−25.59
ICU days	15.46	20.38	−4.92
Costs (2022 USD^a^)			
Cost of Diagnostic Tests	$7,600	$3,800	$+$3,800
Cost of Resource Utilization	$22,254	$26,153	-$3,899
Total Costs	$29,854	$29,953	-$99
Additional Outcomes			
ILI-related Antibiotic Use	183	275	−92
Cost of Antibiotics^b^	$1,096	$1,650	-$554

a Converted using 1 USD: 5 BRL.

b Costs of antibiotic medications are presented here but represent out of pocket costs to the patient rather than costs reimbursed by the private health insurance plan.

The cost of diagnostic testing increased in the combination testing arm, reflecting the higher price of the combination test ($7.6) vs. a standalone COVID-19 test ($3.8). These costs were offset by reductions in HCRU related costs (HCRU costs estimated at $22,254 for combination RDT testing vs. $26,153 for standard practice), resulting in a cost saving of $99.

### Scenario analyses

**Fig. 2:**
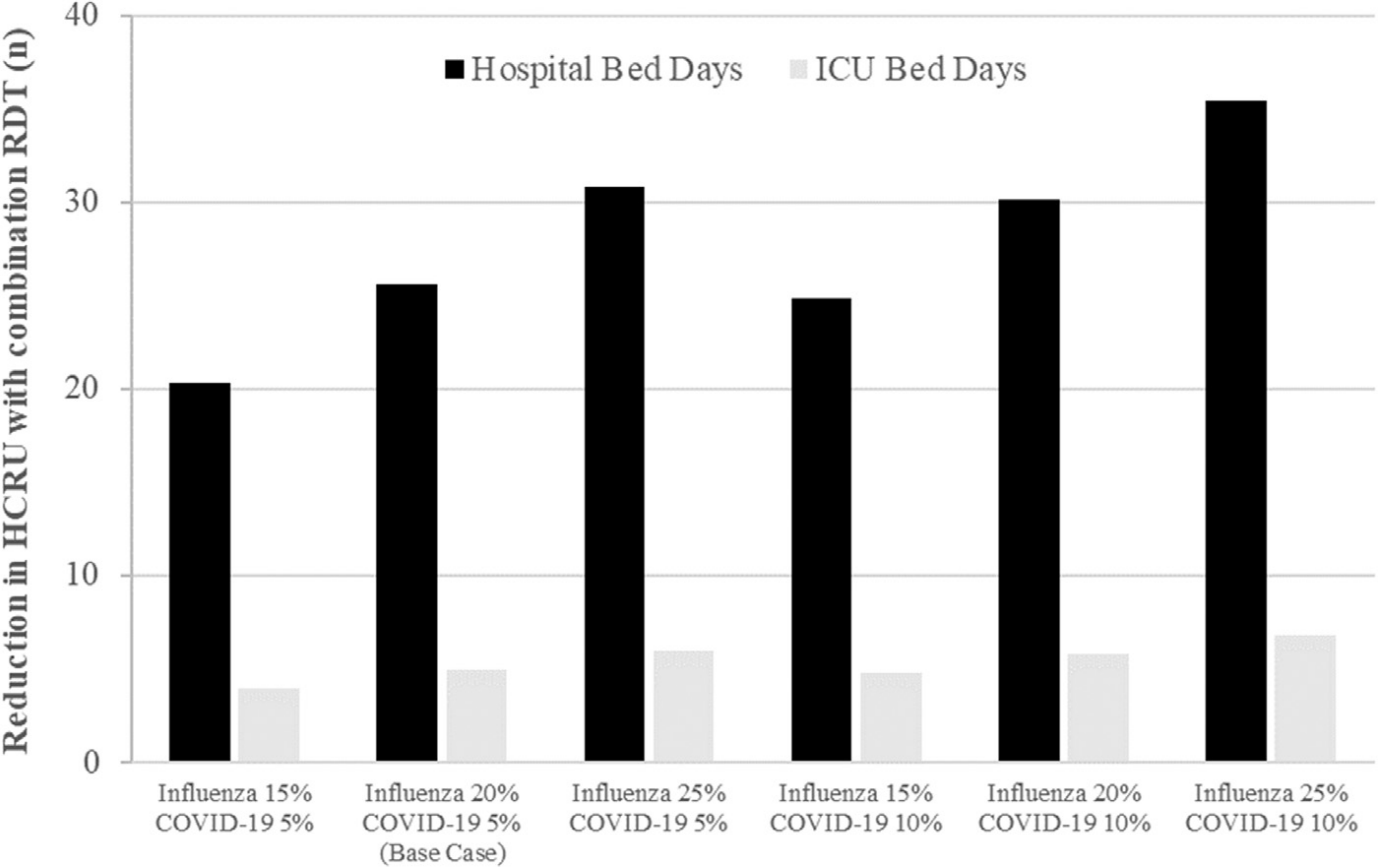
Scenario analyses - impact of influenza and COVID-19 prevalence on HCRU.

Fig. 2 provides results of the scenario analyses. Under all examined scenarios, use of combination RDT testing decreased HCRU compared to standard practice. The impact on hospital bed days ranged from a reduction of 20.3 bed days per 1000 people (15 % influenza prevalence and 5 % COVID-19 prevalence) to a reduction of 35.4 bed days per 1000 people (25 % influenza prevalence and 10 % COVID-19 prevalence). Comparable results were seen for ICU data, with lower prevalence settings resulting in a decrease of 3.9 ICU days per 1000 people and higher prevalence settings in a decrease of 6.8 ICU days per 1000 people. The positive impact on HCRU offset the additional diagnostic costs in all scenarios where influenza prevalence was equal to or greater than 20 % (four of the six scenarios). A full tabulation of results is provided in the supplementary materials (Table S1).

### Sensitivity analyses and additional outcomes

The OWSA (reported in the Supplemental Appendix) indicates that under current model assumptions, model outputs were most sensitive to the cost of the combination RDT diagnostic and the extent to which early diagnosis of influenza is assumed to impact subsequent HCRU.

**Fig. 3:**
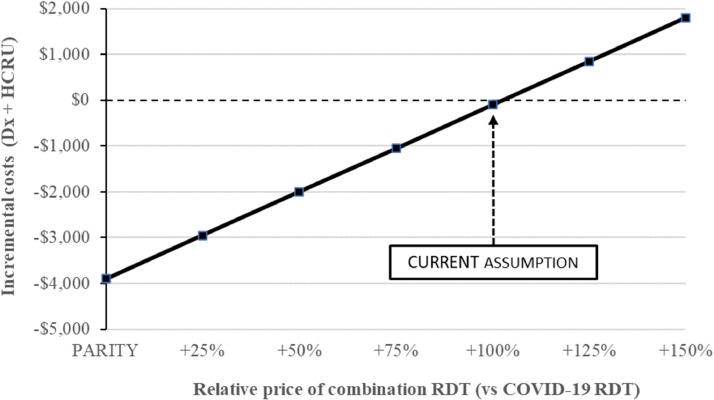
Sensitivity Analysis: Fluctuation in RDT relative costs.

Fig. 3 illustrates how the comparative pricing of a combination RDT relative to an individual COVID-19 RDT would impact results. Our base case found that a combination RDT is cost-saving at twice the price of an individual COVID-19 RDT (100 % increase, cost savings of $99). The figure shows that relative price fluctuations would have a substantial impact on savings.

In the additional analysis where the potential impact of RDT use on prescribing of antibiotics was explored, standard practice (i.e., no RDT for influenza) was estimated to result in 275 unnecessary antibiotic prescriptions in a cohort of 1000 ILI adults, and use of an RDT (in this case the combination RDT) could reduce this to 183 prescriptions. Using a provisional cost of $6 per prescription (equivalent to 30 BRL), this could result in additional savings of $554 (Table 2).

## Discussion

This study developed a novel dual decision tree model to estimate the impact of a COVID-19/influenza RDT for adults presenting with ILI in an ambulatory healthcare setting. In the base case analysis, a switch to a combination COVID-19/influenza RDT compared to current standard practice of presumptive influenza diagnosis and a COVID-19 RDT resulted in fewer missed influenza diagnoses (including co-infection), fewer instances of over-prescribing of antivirals, and reductions in hospital and ICU bed days. In addition, use of the combination RDT was estimated to reduce unnecessary antibiotic prescriptions.

These results are not surprising as they reflect the higher sensitivity and specificity of the RDT compared to clinical judgment for influenza but indicate the broader impact to the healthcare system that may result from a more accurate diagnosis. When the costs of these outcomes are considered, the use of a combination COVID-19/influenza RDT has the potential to result in overall savings to the private health insurer, but also reduced patient out of pocket costs for unnecessary treatment. These savings were the result of the additional costs of the combination RDT being offset by savings associated with reduced HCRU.

The scenario analyses indicate the strong relationship of the model results to disease prevalence, particularly influenza. This is unsurprising, as higher rates of disease will result in more positive cases who are then appropriately treated, reducing their risk of complications. It is important to note however, that the benefits of a combination RDT appear greatest when both influenza and COVID-19 prevalence are high. This scenario represents a greater diagnostic challenge for clinicians as well, since differentiating between infections will be more difficult when both viruses are circulating at high levels.

As noted earlier, this analysis made a simplifying assumption to only consider influenza A, while the combination RDT includes the capability to assess influenza B as well. These results may therefore underestimate the total benefit of a combination COVID-19/influenza RDT, and future research should explore the additional impact of influenza B.

In addition to these established metrics, an exploratory analysis suggested the potential for a 34 % reduction in antibiotic prescriptions through a combination of behavior modification (use of any POCT) and an increase in confirmed positive diagnoses of influenza. These results should be explored further in future studies.

This analysis focuses on short-term outcomes, but it is useful to relate these metrics to potential long-term impacts of improved diagnosis. Through reducing the number of missed diagnoses (fewer false negatives), the likelihood of onward disease transmission is reduced. This suggest that broader use of a combination RDT could positively impact community levels of disease. Dynamic infectious disease modeling approaches could be used to further explore this area.

There were several limitations to this analysis. Metrics were taken from disparate sources, and simplifying assumptions were made for estimating costs across IP and ICU settings. Though defendable, a more systematic approach to sourcing and estimating key model inputs would be helpful, particularly if real world data were available at a local level to validate key model inputs. Estimates for influenza clinical judgment accuracy were obtained from a US study and may not reflect the situation in Brazil. Similarly, treatment effects on the risk of hospitalization for influenza were also obtained from a US study.

The narrow cost perspective applied in this analysis means that some important aspects of testing for COVID-19 and influenza from a personal or societal perspective were not considered. On top of reduced HCRU, benefits of more accurate diagnoses may include reduced absenteeism, and reduction in costs for antiviral medications (typically not reimbursed by Brazilian private insurance plans).

Despite these limitations, we present a robust model framework for estimating the health and cost benefits of use of a dual diagnostic in cases where currently only COVID-19 RDTs are used.

## Conclusions

Based on current model assumptions, moving from usual care (clinical judgment for influenza and COVID-19 testing) to a combination COVID-19/influenza RDT could result in reductions in missed diagnoses, reduced hospital and ICU days, and the potential for overall savings to Brazilian private health insurance plans.

## Conflicts of interest

Ms. Sotak and Ms. Haas report employment by and stock ownership in Abbott. Ms. Lowin and Mr. Wastlund are employees of Vista Health Pte Ltd.

## Appendix Supplementary materials

Image, application 1

## References

[1] Merckx J., Wali R., Schiller I., Caya C., Gore G.C., Chartrand C. (2017). Diagnostic accuracy of novel and traditional rapid tests for influenza infection compared with reverse transcriptase polymerase chain reaction: a systematic review and meta-analysis. Ann Intern Med.

[2] Dugas A.F., Valsamakis A., Atreya M.R., Thind K., Manchego P.A., Faisal A. (2015). Clinical diagnosis of influenza in the ED. Am J Emerg Med.

[3] Stein J., Louie J., Flanders S., Maselli J., Hacker J.K., Drew W.L. (2005). Performance characteristics of clinical diagnosis, a clinical decision rule, and a rapid influenza test in the detection of influenza infection in a community sample of adults. Ann Emerg Med.

[4] World Health Organisation. Coronavirus disease (COVID-19: similarities and differences between COVID-19 and influenza. Available from: https://www.who.int/emergencies/diseases/novel-coronavirus-2019/question-and-answers-hub/q-a-detail/coronavirus-disease-covid-19-similarities-and-differences-with-influenza.

[5] Musuuza J., Watson L., Parmasad V., Putman-Buehler N., Christensen L., Safdar N. (2021). Prevalence and outcomes of co-infection and superinfection with SARS-CoV-2 and other pathogens: a systematic review and meta-analysis. PLoS One.

[6] Hammond J., Leister-Tebbe H., Gardner A., Abreu P., Bao W., Wisemandle W. (2022). Oral nirmatrelvir for high-risk, nonhospitalized adults with COVID-19. N Engl J Med.

[7] Dai Z., Zhang L., Yu Q., Liu L., Yang M., Fan K. (2020). Early administration of oseltamivir within 48 hours after onset of flulike symptoms can reduce the risk of influenza B virus-associated pneumonia in hospitalized pediatric patients with influenza B virus infection. Ped Inf Dis J.

[8] Muthuri S.G., Venkatesan S., Myles P.R., Leonardi-Bee J., Al Khuwaitir T.S.A., Al Mamun A., PRIDE Consortium Investigators (2014). Effectiveness of neuraminidase inhbitors in reducing mortality in patients admitted to hospital with influenza A H1N1pdm9 virus infection: a meta-analysis of individual participant data. Lancet Respir Med.

[9] Adams K., Tastad K.J., Huang S., Ujamaa D., Kniss K., Cummings C. (2022). Prevalence of SARS-CoV-2 and influenza coinfection and clinical characteristics among children and adolescents aged <18 years who were hospitalized or died with influenza - United States, 2021-2022 influenza season. MMWR Morb Mortal Wkly Rep.

[10] Garg I., Gangu K., Shuja H., Agahi A., Sharma H., Bobba A. (2022). COVID-19 and influenza coinfecton outcomes among hospitalized patients in the United States: a propensity matched analysis of National Inpatient Sample. Vaccines.

[11] Swets M.C., Russell C.D., Harrison E.M., Docherty A.B., Lone N., Girvan M. (2022). SARS-CoV-2 coinfection with influenza viruses, respiratory syncytial virus, or adenoviruses. Lancet.

[12] Nott R., Fuller T.L., Brasil P., Nielsen-Saines K. (2022). Out of season influenza during a COVID-19 void in the state of Rio de Janeiro, Brazil: temperature matters. Vaccines.

[13] Faico-Filho K.S., Barbosa G.R., Bellei N. (2022). Peculiar H3N2 outbreak in São Paulo during summer and emergence of the Omicron variant. J Infection.

[14] Stevenson M., Metry A., Messenger M. (2021). Modelling of hypothetical SARS-CoV-2 point of care tests for routine testing in residential care homes: rapid cost-effectiveness analysis. Health Technol Assess.

[15] Stevenson M., Metry A., Messenger M. (2021). Modelling of hypothetical SARS-CoV-2 point-of-care tests on admission to hospital from A&E: rapid cost-effectiveness analysis. Health Technol Assess.

[16] Diel R., Nienhaus A. (2019). Cost-benefit analysis of real-time influenza testing for patients in German emergency rooms. Int J Environ Res Public Health.

[17] Allen A.J., O'Leary R.A., Davis S., Graziadio S., Jones W.S., Simpson A.J. (2018). Cost implications for the NHS of using the Alere™ i Influenza A & B near patient test with nasal swabs. Diagn Progn Res.

[18] Diel R., Nienhaus A. (2022). Point-of-care COVID-19 antigen testing in German emergency rooms - a cost-benefit analysis. Pulmonology.

[19] You J.H.S., Tam L.P., Lee N.L.S. (2017). Cost-effectiveness of molecular point-of-care testing for influenza viruses in elderly patients at ambulatory care setting. PLoS One.

[20] Mac S., O'Reilly R., Adhikari N.K.J., Fowler R., Sander B. (2020). Point-of-care diagnostic tests for influenza in the emergency department: a cost-effectiveness analysis in a high-risk population from a Canadian perspective. PLoS One.

[21] World Health Organization. FluNet summary. Available from: https://www.who.int/tools/flunet/flunet-summary.

[22] Abbott Rapid Diagnostics Jena GmBH. PANBIO™ COVID-19/Flu A&B rapid panel (Nasopharyngeal) instructions for use.

[23] Alosaimi B., Naeem A., Hamed M.E., Alkadi H.S., Alanazi T., Al Rehily S.S. (2021). Influenza co-infection associated with severity and mortality in COVID-19 patients. Virol J.

[24] Crépey P., Boiron L., Araujo R.R., Lopez J.G., Petitjean A., Luna E.J.A. (2020). Impact of quadrivalent influenza vaccines in Brazil: a cost-effectiveness analysis using an influenza transmission model. BMC Public Health.

[25] Rocha J.L.L., Riediger I., Gasparetto J., Tuon F.F. (2023). COVID-19 in real world: survival and medical costs of hospitalized patients in Brazil's first wave. Braz J Infect Dis.

[26] XE.com currency converter. Available from: https://www.xe.com/.

[27] Martinot M., Greigert V., Gravier S. (2019). Positive impact of a point-of-care molecular influenza test in the emergency department during the 2017-2018 seasonal influenza epidemic. Open Forum Inf Dis.

[28] Krantz E.M., Zier J., Stohs E., Ogimi C., Sweet A., Marquis S. (2020). Antibiotic prescribing and respiratory viral testing for acute upper respiratory infections among adult patients at an ambulatory cancer center. Clin Infect Dis.

[29] Egilmezer E., Walker G.J., Bakthavathsalam P., Peterson J.R., Gooding J.J., Rawlinson W. (2018). Systematic review of the impact of point-of-care testing for influenza on the outcomes of patients with acute respiratory tract infection. Rev Med Virol.

[30] O'Connell S., Conlan C., Reidy M. (2020). The impact of point-of-care testing for influenza A and B on patient flow and management in a medical assessment unit of a general hospital. BMC Res Notes.

[31] Tillekeratne L.G., Bodinayake C.K., Nagahawatte A., Vidanagama D., Devasiri V., Arachchi W.K. (2015). Use of rapid influenza testing to reduce antibiotic prescriptions among outpatients with influenza-like illness in southern Sri Lanka. Am J Trop Med Hyg.

[32] Piroth L., Cottenet J., Mariet A.-S., Bonniaud P., Blot M., Tubert-Bitter P. (2021). Comparison of the characteristics, morbidity, and mortality of COVID-19 and seasonal influenza: a nationwide, population-based retrospective cohort study. Lancet Respir Med.

[33] Hui B., Guo H., Zhou P., Shi Z.-L. (2021). Characteristics of SARS-CoV-2 and COVID-19. Nat Rev Microbiol.

[34] Brazilian Ministry of Health. SUS hospital information system (SIH/SUS). Available from: https://datasus.saude.gov.br/acesso-a-informacao/producao-hospitalar-sih-sus/.

[35] Santos H.L.P.C., Maciel F.B.M., Santos Junior G.M., Martins P.C., Lima Prado N.M.B (2021). Public expenditure on hospitalizations for COVID-19 treatment in 2020, in Brazil. Rev Saude Publica.

[36] Miethke-Morais A., Cassenote A., Piva H., Tokunaga E., Cobello V., Gonçalves F.A.R. (2021). COVID-19-related hospital cost-outcome analysis: the impact of clinical and demographic factors. Braz J Infect Dis.

