# Supplementary material for: Cost-consequence analysis of a combined COVID-19/influenza rapid diagnostic test in the Brazilian private healthcare setting
Source: Braz J Infect Dis. 2024 Jul 9;28(4):103840. doi: 10.1016/j.bjid.2024.103840 (PMC11301055; doi:10.1016/j.bjid.2024.103840)
Supplement: Supplementary file 1 [file mmc1.docx]

**BJID-D-24-00004_Supplementary Material**

**Supplementary Material**

**Table S1** Scenario outputs.

| **Prevalence scenario** | **Outcomes avoided with use of a combination RDT** | | | | **Cost impact (net, USD)** |
| --- | --- | --- | --- | --- | --- |
|  | **Over-diagnosis** | **Missed diagnosis** | **Hospital Bed days** | **ICU bed days** |  |
| Influenza 15% COVID-19 5% | 185.05 | 88.25 | 20.33 | 3.91 | $702.54 |
| Influenza 20% COVID-19 5% | 174.05 | 116.25 | 25.59 | 4.92 | -$98.89 |
| Influenza 25% COVID-19 5% | 163.05 | 144.25 | 30.86 | 5.93 | -$900.32 |
| Influenza 15% COVID-19 10% | 183.10 | 92.50 | 24.88 | 4.78 | $9.38 |
| Influenza 20% COVID-19 10% | 172.10 | 120.50 | 30.15 | 5.80 | -$792.05 |
| Influenza 25% COVID-19 10% | 161.10 | 148.50 | 35.41 | 6.81 | -$1593.48 |

**Figure S1** Sensitivity analysis: tornado diagram. KEY. HCRU, Healthcare Related Resource Use; iA, influenza A; RR, Risk Reduction; TP, True Positive.


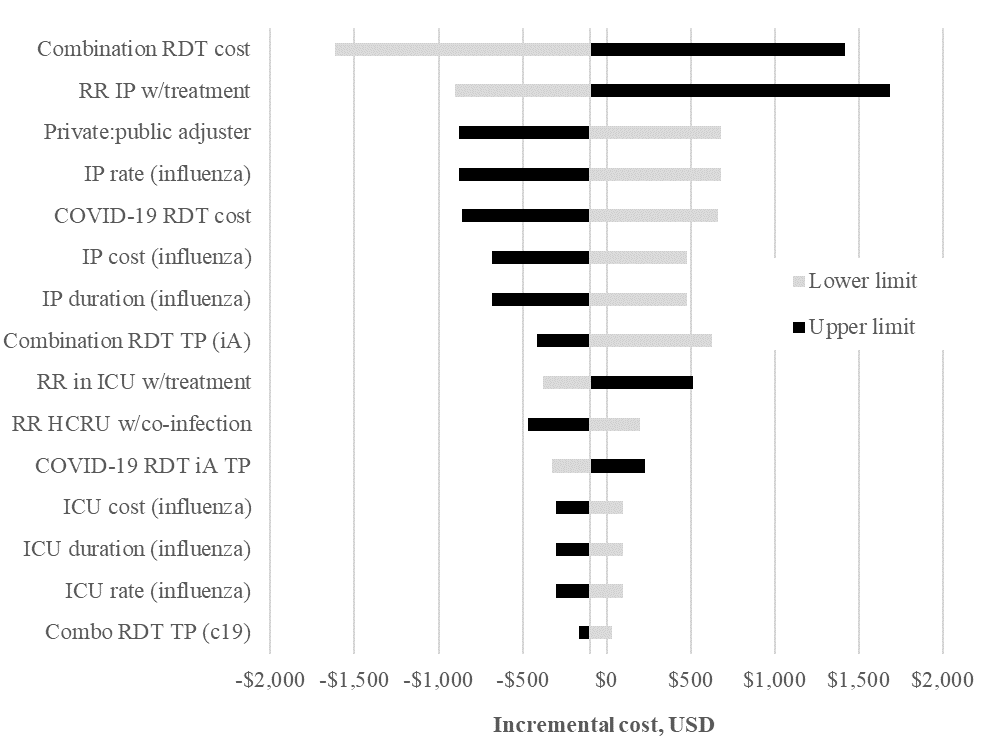


**CHEERS 2022 Checklist.**

| **Topic** | **Nº** | **Item** | **Location where item is reported** |
| --- | --- | --- | --- |
| **Title** |  |  |  |
|  | 1 | Identify the study as an economic evaluation and specify the interventions being compared. | Introduction |
| **Abstract** |  |  |  |
|  | 2 | Provide a structured summary that highlights context, key methods, results, and alternative analyses. | Abstract |
| **Introduction** |  |  |  |
| **Background and objectives** | 3 | Give the context for the study, the study question, and its practical relevance for decision making in policy or practice. | Introduction |
| **Methods** |  |  |  |
| **Health economic analysis plan** | 4 | Indicate whether a health economic analysis plan was developed and where available. | Not available |
| **Study population** | 5 | Describe characteristics of the study population (such as age range, demographics, socioeconomic, or clinical characteristics). | Materials and Methods ‒ Decision Problem |
| **Setting and location** | 6 | Provide relevant contextual information that may influence findings. | Materials and Methods ‒ Economic Model |
| **Comparators** | 7 | Describe the interventions or strategies being compared and why chosen. | Materials and Methods ‒ Economic Model |
| **Perspective** | 8 | State the perspective(s) adopted by the study and why chosen. | Materials and Methods ‒ Model Inputs |
| **Time horizon** | 9 | State the time horizon for the study and why appropriate. | Materials and Methods ‒ Model Inputs |
| **Discount rate** | 10 | Report the discount rate(s) and reason chosen. | As a short-term time, horizon was used, discounting was not applied |
| **Selection of outcomes** | 11 | Describe what outcomes were used as the measure(s) of benefit(s) and harm(s). | Materials and Methods ‒ Economic Model |
| **Measurement of outcomes** | 12 | Describe how outcomes used to capture benefit(s) and harm(s) were measured. | Materials and Methods ‒ Economic Model |
| **Valuation of outcomes** | 13 | Describe the population and methods used to measure and value outcomes. | Materials and Methods ‒ Economic Model |
| **Measurement and valuation of resources and costs** | 14 | Describe how costs were valued. | Materials and Methods ‒ Model Inputs |
| **Currency, price date, and conversion** | 15 | Report the dates of the estimated resource quantities and unit costs, plus the currency and year of conversion. | Materials and Methods ‒ Model Inputs |
| **Rationale and description of model** | 16 | If modelling is used, describe in detail and why used. Report if the model is publicly available and where it can be accessed. | Materials and Methods ‒ Economic Model |
| **Analytics and assumptions** | 17 | Describe any methods for analyzing or statistically transforming data, any extrapolation methods, and approaches for validating any model used. | Materials and Methods ‒ Economic Model and Model Inputs |
| **Characterizing heterogeneity** | 18 | Describe any methods used for estimating how the results of the study vary for subgroups. | No subgroups were considered |
| **Characterizing distributional effects** | 19 | Describe how impacts are distributed across different individuals or adjustments made to reflect priority populations. | N/A |
| **Characterizing uncertainty** | 20 | Describe methods to characterize any sources of uncertainty in the analysis. | Materials and Methods ‒ Sensitivity Analyses and Figure 4 |
| **Approach to engagement with patients and others affected by the study** | 21 | Describe any approaches to engage patients or service recipients, the general public, communities, or stakeholders (such as clinicians or payers) in the design of the study. | No approaches for patient or general public engagement were made for this study |
| **Results** |  |  |  |
| **Study parameters** | 22 | Report all analytic inputs (such as values, ranges, references) including uncertainty or distributional assumptions. | Table 1 |
| **Summary of main results** | 23 | Report the mean values for the main categories of costs and outcomes of interest and summarize them in the most appropriate overall measure. | Results and Table 2 |
| **Effect of uncertainty** | 24 | Describe how uncertainty about analytic judgments, inputs, or projections affect findings. Report the effect of choice of discount rate and time horizon, if applicable. | Results ‒ Sensitivity Analyses and Figure 4 |
| **Effect of engagement with patients and others affected by the study** | 25 | Report on any difference patient/service recipient, general public, community, or stakeholder involvement made to the approach or findings of the study | No approaches for patient or general public engagement were made for this study |
| **Discussion** |  |  |  |
| **Study findings, limitations, generalizability, and current knowledge** | 26 | Report key findings, limitations, ethical or equity considerations not captured, and how these could affect patients, policy, or practice. | Discussion |
| **Other relevant information** |  |  |  |
| **Source of funding** | 27 | Describe how the study was funded and any role of the funder in the identification, design, conduct, and reporting of the analysis | Reported in funding section |
| **Conflicts of interest** | 28 | Report authors conflicts of interest according to journal or International Committee of Medical Journal Editors requirements. | Reported in funding section |

From: Husereau D, Drummond M, Augustovski F, et al. Consolidated Health Economic Evaluation Reporting Standards 2022 (CHEERS 2022) Explanation and Elaboration: A Report of the ISPOR CHEERS II Good Practices Task Force. Value Health 2022;25. doi:10.1016/j.jval.2021.10.008.
